# In Vitro Activity of Sertraline, an Antidepressant, Against Antibiotic-Susceptible and Antibiotic-Resistant *Helicobacter pylori* Strains

**DOI:** 10.3390/pathogens8040228

**Published:** 2019-11-10

**Authors:** Paweł Krzyżek, Roman Franiczek, Barbara Krzyżanowska, Łukasz Łaczmański, Paweł Migdał, Grażyna Gościniak

**Affiliations:** 1 Department of Microbiology, Faculty of Medicine, Wroclaw Medical University, Wroclaw 50-368, Poland; roman.franiczek@umed.wroc.pl (R.F.); barbara.krzyzanowska@umed.wroc.pl (B.K.); grazyna.gosciniak@umed.wroc.pl (G.G.); 2 Hirszfeld Institute of Immunology and Experimental Therapy, Polish Academy of Sciences, Wroclaw 53-114, Poland; lukasz@diagmol.com; 3 Department of Environment, Hygiene and Animal Welfare, Wroclaw University of Environmental and Life Sciences, Wroclaw 51-630, Poland; pawel.migdal@upwr.edu.pl

**Keywords:** coccoid forms, checkerboard assay, selective serotonin reuptake inhibitor, time-killing assay

## Abstract

Antibiotic resistance of *Helicobacter pylori*, a spiral bacterium associated with gastric diseases, is a topic that has been intensively discussed in last decades. Recent discoveries indicate promising antimicrobial and antibiotic-potentiating properties of sertraline (SER), an antidepressant substance. The aim of the study, therefore, was to determine the antibacterial activity of SER in relation to antibiotic-sensitive and antibiotic-resistant *H. pylori* strains. The antimicrobial tests were performed using a diffusion-disk method, microdilution method, and time-killing assay. The interaction between SER and antibiotics (amoxicillin, clarithromycin, tetracycline, and metronidazole) was determined by using a checkerboard method. In addition, the study was expanded to include observations by light, fluorescence, and scanning electron microscopy. The growth inhibition zones were in the range of 19–37 mm for discs impregnated with 2 mg of SER. The minimal inhibitory concentrations (MICs) and minimal bactericidal concentrations (MBCs) counted for 2–8 µg/mL and 4–8 µg/mL, respectively. The time-killing assay showed the time-dependent and concentration-dependent bactericidal activity of SER. Bacteria exposed to MBCs (but not sub-MICs and MICs ≠ MBCs) underwent morphological transformation into coccoid forms. This mechanism, however, was not protective because these cells after a 24-h incubation had a several-fold reduced green/red fluorescence ratio compared to the control. Using the checkerboard assay, a synergistic/additive interaction of SER with all four antibiotics tested was demonstrated. These results indicate that SER may be a promising anti-*H. pylori* compound.

## 1. Introduction

The presence of spiral microorganisms inhabiting the gastric mucosa was noticed more than 100 years ago, but the lack of a way to isolate these microorganisms using standard culture methods probably reduced interest in this topic [1]. A key moment was the isolation of a spiral, Gram-negative rod, known today as *Helicobacter pylori*, which was obtained from gastric biopsies by Barry Marshall and Robin Warren in 1983. The discovery of the relationship between the presence of this bacterium and the development of gastric ulcers contributed to the flourishing of research carried on this microorganism. As a result, Barry Marshall and Robin Warren were crowned with the Nobel Prize in 2005 [2]. Subsequent progression in research on *H. pylori* has shown the involvement of this bacterium in the development of gastric diseases, including gastritis, gastric and duodenal ulcers, gastric cancers, and gastric mucosa-associated lymphoid tissue lymphomas [3]. Currently, the detection of *H. pylori* in patients, regardless of the presence of symptoms, is an indication for antibiotic therapy. This recommendation was administered during three independent consensus conferences in Brazil, Kyoto, and Maastricht [4,5,6].

An excessive consumption of antibiotics in medicine, veterinary, and agricultural industries is a global problem that has contributed to the rapid increase in antibiotic resistance of many pathogenic microorganisms, including *H. pylori* [7]. This topic has been intensively discussed in recent years. It has been noted that antibiotic resistance in *H. pylori* reached an alarmingly high level with the prevalence of strains resistant to clarithromycin, metronidazole, and/or levofloxacin exceeding 15% within World Health Organization-supervised areas [8]. Nowadays, there are many antibiotic-based therapies against *H. pylori*; however, they have several important limitations. These include resistance spreading, microflora disruption, and the presence of side effects (diarrhea, nausea, vomiting, or lower abdominal pain), all of which affect patient non-adherence and, as a consequence, cause therapeutic failure [9,10,11]. Given the problems arising from the use of current antibiotic therapies, there is a high need to find new substances with an anti-*H. pylori* activity, which could be used in monotherapy, as well as an adjuvant/synergistic therapy [9].

One group of compounds with promising antimicrobial properties are selective serotonin reuptake inhibitors (SSRIs) [12]. Classically, these substances are used as the first line therapy for people suffering from depression, which is associated with the ability of SSRIs to affect the level of neurotransmitters in the brain, with particular emphasis on serotonin [13]. SSRIs effectively block the reuptake of this neurohormone from synapses in the central nervous system, resulting in enhanced serotonergic transmission and improved patient well-being [14]. In addition to the beneficial psychoactive properties, the researchers’ attention was drawn to the antimicrobial activity of SSRIs, which was first demonstrated in the 1990s [12]. Over time, the number of reports confirming the antimicrobial activity of SSRIs increased significantly, showing antifungal [15,16,17,18,19,20,21,22,23,24,25], antiparasitic [26,27,28,29], and antibacterial [30,31,32,33,34,35,36] properties. These studies indicated that the substance with the highest activity among members of this group is sertraline (SER) [24,25,28,30,31]. In addition to the strong antimicrobial properties of SER, the ability to potentiate antibiotic activity has also been demonstrated [33,36,37]. This feature seems particularly interesting in the context of antibacterial activity against antibiotic-resistant *H. pylori* strains.

The purpose of this original article was to determine the antimicrobial activity of SER against antibiotic-sensitive and antibiotic-resistant *H. pylori* strains, both alone and in combination with clinically used antibiotics. The research has been extended by an analysis of morphology of bacterial cells exposed to different concentrations of SER.

## 2. Materials and Methods

### 2.1. Bacterial Strains and Culture Conditions

The research was carried out using 52 *H. pylori* strains (50 clinical strains used previously, and two reference strains, Tx30a (ATCC 51932) and J99 (ATCC 700824)) [38]. Clinical strains belong to the collection of microorganisms of the Department of Microbiology at Wroclaw Medical University. *H. pylori* J99 was obtained by the courtesy of Dr. A. Zawilak-Pawlik (Hirszfeld Institute of Immunology and Experimental Therapy, Polish Academy of Sciences, Poland), while *H. pylori* Tx30a was obtained from the American Type Culture Collection (ATCC). The isolated strains were identified as *H. pylori* based on the spiral morphology and positive reactions of catalase, oxidase, and urease tests. These strains have been categorized as antibiotic-resistant based on the EUCAST recommendations, i.e., amoxicillin (AMX) > 0.125 µg/mL, clarithromycin (CLR) > 0.5 µg/mL, tetracycline (TET) > 1 µg/mL, and metronidazole (MTZ) > 8 µg/mL [39]. 

Bacterial strains were stored in Tryptic Soy Broth (TSB) (Oxoid, Le Pont de Claix, France) with 15% glycerol at −70 °C until needed. After reviving, bacteria were seeded on Columbia agar (Difco, Lublin, Poland) with 7% hemolyzed horse blood (CA + HB) and incubated for three days under microaerophilic conditions (Genbox microaer kits, BioMerieux, Marcy I’Etoile, France) at 37 °C [38,40]. The grown bacteria were passaged on CA + HB and again incubated under previously mentioned conditions for the next three days.

### 2.2. Disk-Diffusion Method

Suspensions of tested *H. pylori* strains (4 McFarland units, approx. 10^8^ CFU/mL) were prepared using Brain Heart Infusion broth (BHI, Oxoid) with the addition of 7% foetal calf serum (Gibco) (BHI + FCS) [38]. Each of them were sown by cotton swabs on the CA + HB agar surface (5 × 10^6^ CFU/mL). The 20 µL of three SER solutions (100 mg/mL, 50 mg/mL, and 10 mg/mL) were spotted on paper disks placed on the surface of each CA + HB agar, obtaining 2 mg/disk, 1 mg/disk and 0.2 mg/disk, respectively. SER was dissolved in dimethyl sulfoxide (DMSO, Sigma-Aldrich) with a final concentration not exceeding 1% (*v/v*). Disks with AMX (Oxoid, 25 µg/disk) and 1% DMSO were positive and negative controls, respectively. The agar plates with bacteria were incubated for three days in microaerophilic conditions at 37 °C. After this time, the growth inhibition zones were read.

### 2.3. MIC/MBC Determination

Determination of minimal inhibitory concentrations (MICs) and minimal bactericidal concentrations (MBCs) was carried out using the microdilution method in 12-well titration plates (Bionovo, Legnica, Poland) [38]. Each well of the microplate contained a 1 mL of bacterial suspension (approx. 10^7^ CFU/mL) in BHI + FCS broth and a SER concentration gradient (0.25–16 µg/mL). The microdilution plates were incubated for three days under microaerophilic conditions at 37 °C with shaking (100 rpm). The positive and negative controls were microplate wells containing bacteria without SER and culture medium without bacteria, respectively. 

The MIC was considered to be the lowest concentration at which no bacterial growth in BHI + FCS broth was detected [41]. The MBC value was considered to be the lowest concentration at which no bacterial growth was observed after spotting bacterial suspensions from each well of the microtiter plate onto CA + HB agar [41].

### 2.4. Checkerboard Assay

Synergism of antibacterial activity of SER was determined in combination with AMX (Sigma-Aldrich), CLR (Sigma-Aldrich), TET (Sigma-Aldrich), and MTZ (Sigma-Aldrich) using the checkerboard method. The experiment was performed using six 12-well microtitration plates forming a 72-well panel [38]. Concentration gradients of SER and the selected antibiotic were located within the external wells of the x and y-axes, respectively. The remaining wells were filled with graded, different concentrations of both compounds. Additionally, in each well, a bacterial suspension corresponding to approx. 10^7^ CFU/mL was present. After completing all procedures, the panel of plates was incubated for three days under microaerophilic conditions at 37 °C with shaking (100 rpm).

On the basis of the obtained results, the FIC index (fractional inhibitory concentration, i.e., MIC of substance A in combination/MIC of substance A separately + MIC of substance B in combination/MIC of substance B separately) was calculated. The FIC index values were used to categorize interactions as: ≤0.5 = synergistic, ˃0.5 to ≤1 = additive, ˃1 to ˂4 = neutral and ≥4 = antagonistic [42,43,44].

### 2.5. Time-Killing Assay

Determination of the bacterial culturability at specific time intervals after exposure to SER was conducted based on a method developed by Brown and Jiang [45] with minor modifications. Culture conditions, culture media, and bacterial optical density were similar with those used in the MIC/MBC determination (except for using 2 mL of BHI + FCS broth). The survival of bacteria treated with different concentrations of SER (4× MIC, 2× MIC, MIC and ½× MIC) and in the absence of SER (control) was determined at selected time points (0 h, 1 h, 2 h, 4 h, 6 h, 8 h, and 24 h). The number of *H. pylori* colonies grown was counted and presented in the form of log_10_ CFU/mL.

### 2.6. Light Microscopy

Bacterial morphology was determined during the checkerboard method and time-killing assays [40]. Each time, 50 μL of bacterial suspension was dripped onto slides from each concentration and strain tested, and stained using the Gram’s method. The study was conducted under an Olympus BX50 microscope (Olympus Optical, Tokyo, Japan), using an ×100 immersion lens.

### 2.7. Fluorescence Microscopy

To determine the viability of *H. pylori* strains when assessing the survival at specific time points, the study was expanded to include fluorescence analysis using Live/Dead staining kit (L7012, ThermoFisher, Waltham, MA, USA) [38]. Slides were viewed under an Olympus BX51 microscope (Olympus Optical, Tokyo, Japan), working with a ×10 lens. Using the ImageJ, the green and red fluorescence of regions of interests (ROIs) (50 ROIs/tested sample) was determined and presented as the mean green/red fluorescence ratio. The fluorescence intensity of SYTO9 (green fluorescent dye) and propidium iodide (red fluorescent dye) was measured at emission levels of 530 nm and 640 nm, respectively.

### 2.8. Scanning Electron Microscopy

Bacteria treated with certain concentrations of SER were fixed by adding a 2.5% glutaraldehyde solution (Sigma-Aldrich) [38,40]. After 24 h of fixing, bacteria were centrifuged at 600 g for 5 min and washed three times in 0.1 M cacodylate buffer (Sigma-Aldrich), centrifuging after each wash at 600 g for 5 min. Bacteria were run through the ethanol series (10%, 30%, 50%, 70%, 90%, and 99.8%). After carrying out the ethanol series, the specimens were sprayed with carbon (15 nm) and observed under a scanning electron microscope (Auriga 60, Zeiss, Oberkochen, Germany), using the beam voltage equal to 2 kV and the working distance of 5 mm.

### 2.9. Statistical Analysis

Differences in the activity of SER between *H. pylori* strains during the disk-diffusion method and broth dilution method were assessed by the Kruskal–Wallis and the Mann–Whitney *U* test, respectively. The impact of SER on the culturability of *H. pylori* was checked using the Kaplan-Meier method and the Wilcoxon test. Categorical data were analyzed by the chi-square Pearson test. The significance was considered when *p* ˂ 0.05.

## 3. Results

### 3.1. Disk-Diffusion Method

**Figure 1:**
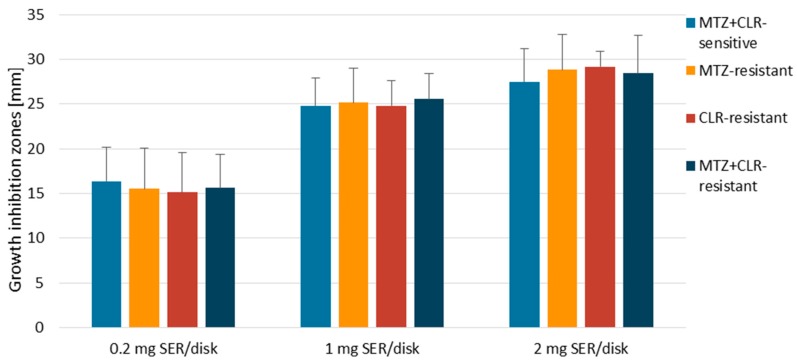
The zones of inhibition (mm) produced by disks with 0.2 mg, 1 mg, and 2 mg of sertraline (SER) against clinical and reference *H. pylori* strains. Amoxicillin (AMX) was a positive control of the study, for which the growth inhibition zones were in the range of 58.5–70.5 mm. The negative control was a 1% DMSO solution (*v/v*) that did not cause the appearance of the growth inhibition zone in all tested *H. pylori* strains (6 mm). Abbreviations: MET, Metronidazole; CLR, Clarithromycin.

During the first stage of the study, the activity of SER was screened against *H. pylori* strains using the disk-diffusion method. It was noticed that the obtained growth inhibition zones were directly proportional to the dose used and accounted for 10–25 mm, 17–33 mm, and 19–37 mm for 0.2 mg, 1 mg, and 2 mg per disk, respectively (Figure 1). Antibiotic resistance of *H. pylori* strains did not determine the size of the growth inhibition zones produced by SER (*p* > 0.05, Supplementary Tables S1–S4). The observed activity of this substance was high, but lower than that of the control antibiotic (AMX).

### 3.2. Determination of MICs and MBCs

The minimal inhibitory concentrations (MICs) and minimal bactericidal concentrations (MBCs) counted for 2–8 µg/mL and 4–8 µg/mL, respectively (Table 1). Among the strains tested, the most sensitive to SER was *H. pylori* 7556 (CLR-resistant) with MIC = 2 µg/mL and MBC = 4 µg/mL, while the least susceptible was *H. pylori* 7471 (antibiotic-sensitive) with MIC and MBC equal to 8 µg/mL. Furthermore, CLR-resistant and double-resistant *H. pylori* strains had lower MIC values (2–4 µg/mL) than clinical, antibiotic-susceptible *H. pylori* strains with MICs of 4–8 µg/mL. Despite these differences in sensitivity to SER, again, this effect was independent of the antibiotic resistance pattern (*p* > 0.05). For all strains, MBC/MIC ratios were equal to ≤4, indicating the bactericidal activity of SER against *H. pylori* (Table 1).

### 3.3. Time-Killing Assay

Initial experiments assessed the bactericidal activity of SER. The effect of this substance on the culturability, viability, and morphology of bacteria over time was estimated afterwards. These parameters were determined relative to two reference *H. pylori* strains (Tx30a and J99).

**Figure 2:**
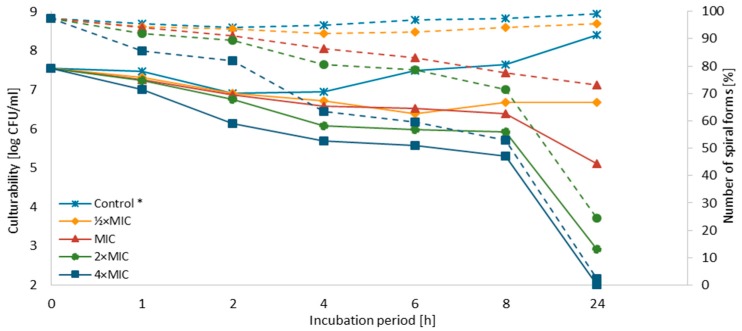
Effect of sertraline (SER) on the culturability and morphology of *H. pylori* Tx30a during the incubation period. *: The number of spiral forms was marked with dotted lines, while the culturability was indicated by continuous lines.

**Figure 3:**
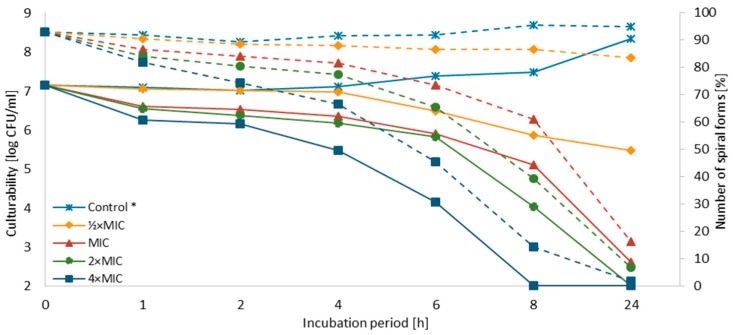
Effect of sertraline (SER) on the culturability and morphology of *H. pylori* J99 during the incubation period. *: The number of spiral forms was marked with dotted lines, while the culturability was indicated by continuous lines.

The concentration-dependent and time-dependent bactericidal effect of SER against both strains was demonstrated (Figure 2 and Figure 3). For *H. pylori* J99, a decrease in the culturability below the detection threshold (<100 CFU/mL, 10^2.00^) was observed after 8 h and 24 h for 4× MIC and 2× MIC, respectively. For MIC, the number of culturable cells after a one-day exposure to SER was close to the detection threshold (400 CFU/mL, 10^2.59^), suggesting that these concentrations are bactericidal against *H. pylori* J99 (MIC = MBC). For *H. pylori* Tx30a, a reduction in the culturability below the detection threshold after a 24-h incubation was only seen for 4× MIC. A one-day exposure of this strain to 2× MIC, however, also resulted in a significant decline of this parameter (800 CFU/mL; 10^2.9^). For both strains, the first 4 h of incubation with SER were associated with a mild decrease in the culturability (reduction log_10_ CFU/mL < 1), except to 4× MIC. In subsequent hours a linear decline in the culturability was noted. For *H. pylori* Tx30a, it was in the range of 10^6.39^–10^5.57^ and 10^6.66^–10^5.29^ for ½× MIC–4× MIC after 6 h and 8 h, respectively (Figure 2 and Supplementary Table S5). In the case of *H. pylori* J99, it was more dynamic with values of 10^6.48^–10^4.15^ and 10^5.86^–10^2.00^ after 6 h and 8 h, respectively (Figure 3 and Supplementary Table S6).

**Figure 4:**
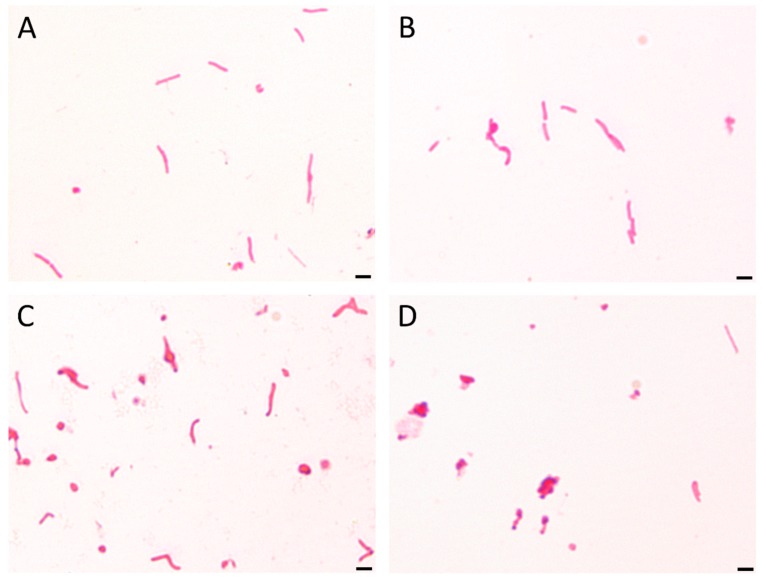
The light microscopy of *H. pylori* cells during the incubation with sertraline (SER). The representative microscopic images of *H. pylori* cells during the incubation with the MBC of SER after (**A**) 0 h, (**B**) 4 h, (**C**) 8 h, and (**D**) 24 h show a time-dependent decrease in the number of spiral forms with an inversely proportional increase in the number of coccoid forms. The scale bar in the light microscopy is 2 μm.

**Figure 5:**
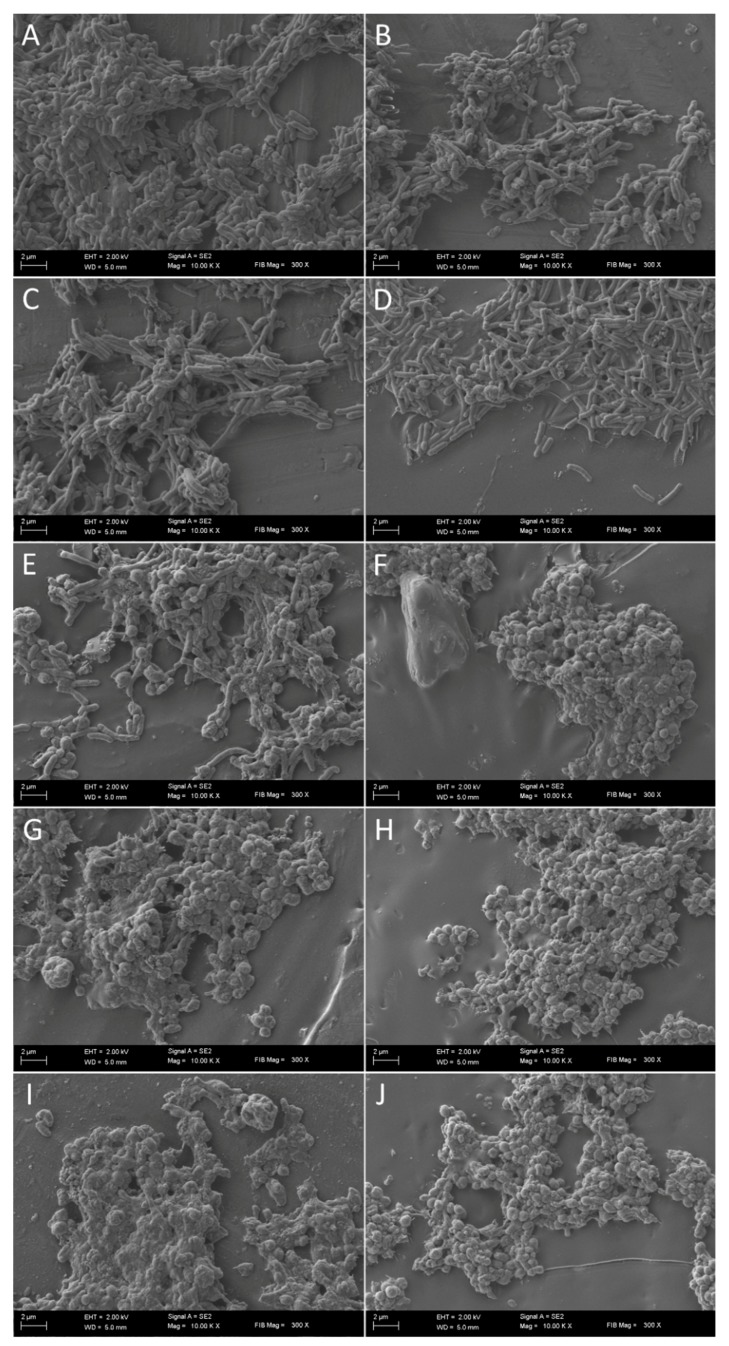
Scanning electron microscopy images of *H. pylori* Tx30a (**A, C, E, G, I**) and J99 (**B, D, F, H, J**) cells without sertraline (SER) (**A, B**) and with ½× MIC of SER (**C, D**), seen mainly as spiral forms, and treated with 2× MIC (**G, H**) and 4× MIC (**I, J**), in which coccoid forms predominate. Cells exposed to MIC of SER differ in a strain-dependent manner with *H. pylori* Tx30a being mainly spiral-shaped (**E**), whereas *H. pylori* J99 in a spherical form (**F**).

It has been observed that the culturability is directly related to the number of spiral forms, because its reduction was accompanied by a decrease in the number of these morphological forms with an inversely proportional increase of spherical forms (Figure 2, Figure 3 and Figure 4). In control samples, untreated with SER, the spiral morphotype was dominant throughout the entire experiment (90–99%). Analyzing the differences in the number of spiral forms between the tested strains, statistical differences were noticed in an 8-h (*p* = 0.0007) and a 24-h (*p* < 0.0000) incubation. The number of spiral forms between control samples and these treated with SER were statistically significantly different starting from a 1-h incubation for *H. pylori* T30a (*p* < 0.0000, except for a 2-h culture with *p* < 0.05) and a 2-h incubation for *H. pylori* J99 (at 2-h, *p* < 0.05, then *p* < 0.0005). For *H. pylori* Tx30a, the number of spiral forms after a one-day exposure to SER counted for 95.5%, 73%, 24.5%, and 2.5% for ½× MIC, MIC, 2× MIC, and 4× MIC, respectively (Figure 2 and Supplementary Table S7). While for *H. pylori* J99, after a 24-h incubation, 83.5, 16.5%, 7%, and 2% spiral forms were shown after exposure to ½× MIC, MIC, 2× MIC, and 4× MIC, respectively (Figure 3 and Supplementary Table S8). The morphological transformation of both *H. pylori* strains was then confirmed using a scanning electron microscope (Figure 5). In control and ½× MIC samples, the spiral morphotype was dominant in both (Figure 5A–D). Convergence of observations was also made for cells exposed to 2× MIC and 4× MIC of SER, for which the coccoid morphotype was predominant (Figure 5G–J). The difference was demonstrated for MICs, because, similarly to observations using light microscopy, it was noticed that *H. pylori* Tx30a appeared mainly in a spiral-shaped form (Figure 5E), whereas *H. pylori* J99 was spherical (Figure 5F). These observations correlate with MBC values, which for *H. pylori* Tx30a are equal to 2× MIC (MIC ≠ MBC), while for *H. pylori* J99 MIC = MBC (Table 1).

**Figure 6:**
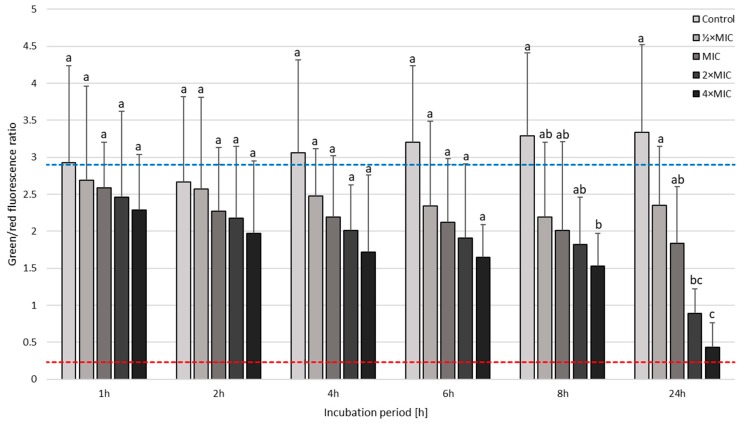
The fluorescence microscopy analysis of the viability during the incubation of *H. pylori* Tx30a with sertraline (SER) in time. The blue top and red bottom lines indicate the positive (a 0-h incubation) and negative controls (a 1-h treatment with 70% ethanol), respectively. Columns with the same subscript letters are not significantly different from each other (*p* > 0.05).

**Figure 7:**
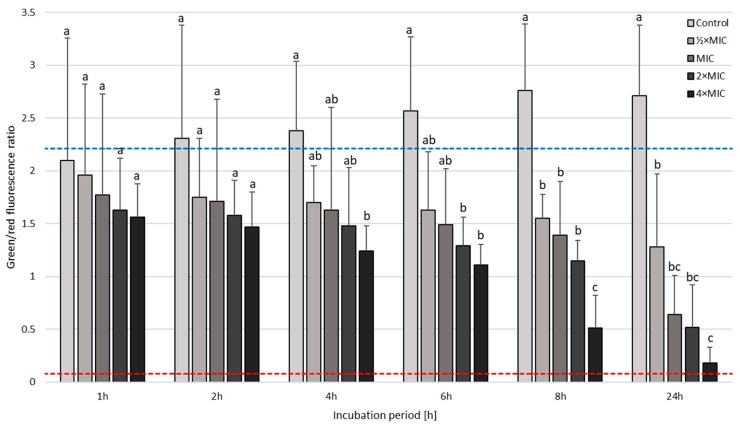
The fluorescence microscopy analysis of the viability during the incubation of *H. pylori* J99 with sertraline (SER) in time. The blue top and red bottom lines indicate the positive (a 0-h incubation) and negative controls (a 1-h treatment with 70% ethanol), respectively. Columns with the same subscript letters are not significantly different from each other (*p* > 0.05).

The next step in the analysis of SER-treated *H. pylori* strains was the determination of the viability using a fluorescence microscope (Figure 6 and Figure 7). This investigation aimed to determine whether coccoid forms, naturally having a higher tolerance to antimicrobial substances, are viable. For both strains tested, differences in the mean green/red fluorescence were statistically significant starting from a 1-h incubation (p < 0.0000). In the case of *H. pylori* Tx30a, a 24-h incubation with MIC, 2× MIC, and 4× MIC of SER resulted in a 2-fold, 4-fold, and 8-fold reduction in the mean green/red fluorescence compared to the control, respectively (Figure 6 and Supplementary Figure S1 and Table S9). A more intense decline in the viability was observed in *H. pylori* J99, i.e., a 4-fold, 5-fold, and 15-fold reduction in the mean green/red fluorescence relative to the control when using MIC, 2× MIC, and 4× MIC, respectively (Figure 7 and Supplementary Figure S2 and Table S10). In addition, the analysis of changes in this parameter within given concentrations at various time points showed statistically significant decreases in green fluorescence over the course of the experiment for all concentrations tested against *H. pylori* J99 (*p* < 0.0000). On the other hand, for *H. pylori* Tx30a, statistical differences were only shown for 2× MIC and 4× MIC (*p* < 0.0000), while for MIC a tendency to reduce over time was seen (*p* = 0.077).

### 3.4. Checkerboard Assay

**Figure 8:**
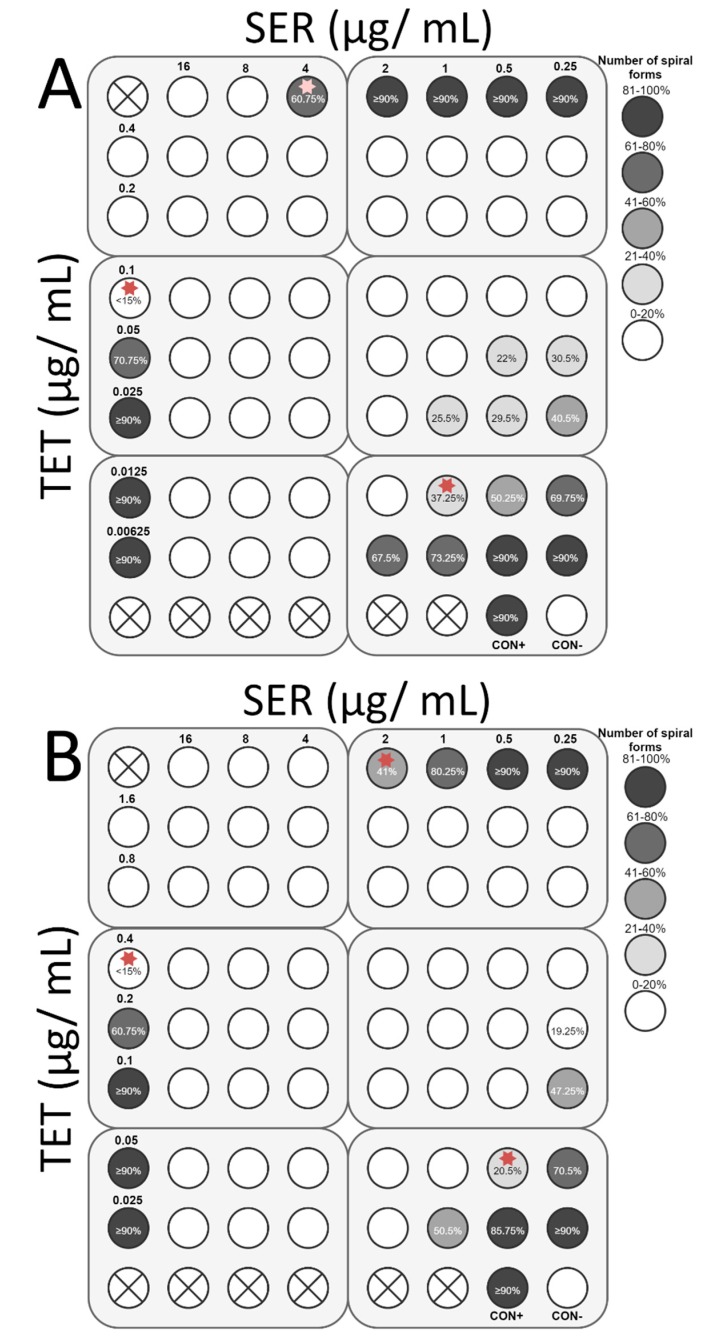
The antibacterial and morphological effect of sertraline (SER), tetracycline (TET), and combinations of both against *H. pylori* Tx30a and 7143 strains. The existence of interaction in the antimicrobial activity of SER with TET was determined against the reference, antibiotic-susceptible *H. pylori* Tx30a (**A**) and the clinical, double-resistant *H. pylori* 7143 strain (**B**). The white circles indicate wells in which the number of spiral forms was ≤15%, the while white circles with a cross in the middle indicate empty wells. Using red asterisks, the wells with the MICs of the tested substances were marked, whereas in the case of the interactions verification, they indicate the lowest FIC. Abbreviations: Sertraline, SER; Tetracycline, TET; Positive control, CON+; Negative control, CON-.

**Figure 9:**
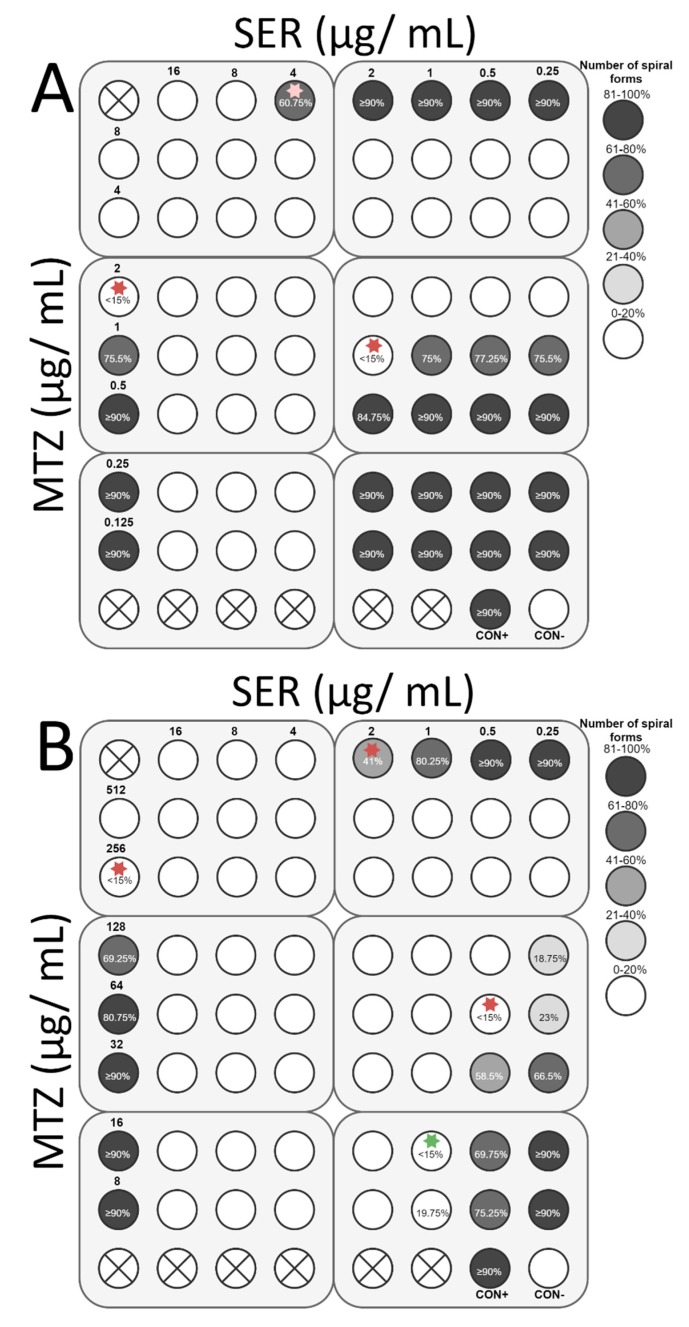
The antibacterial and morphological effect of sertraline (SER), metronidazole (MTZ), and combinations of both against *H. pylori* Tx30a and 7143 strains. The existence of interaction in the antimicrobial activity of SER with MTZ was determined against the reference, antibiotic-susceptible *H. pylori* Tx30a (**A**) and the clinical, double-resistant *H. pylori* 7143 strain (**B**). The white circles indicate wells in which the number of spiral forms was ≤15%, while the white circles with a cross in the middle indicate empty wells. Using red asterisks, the wells with the MICs of the tested substances were marked, whereas in the case of the interactions verification, they indicate the lowest FIC. The green asterisk marked the place where the additive interaction was shown (FIC = 0.506), while the MTZ concentration was significantly reduced (16-fold). Abbreviations: Sertraline, SER; Metronidazole, MTZ; Positive control, CON+; Negative control, CON-.

**Figure 10:**
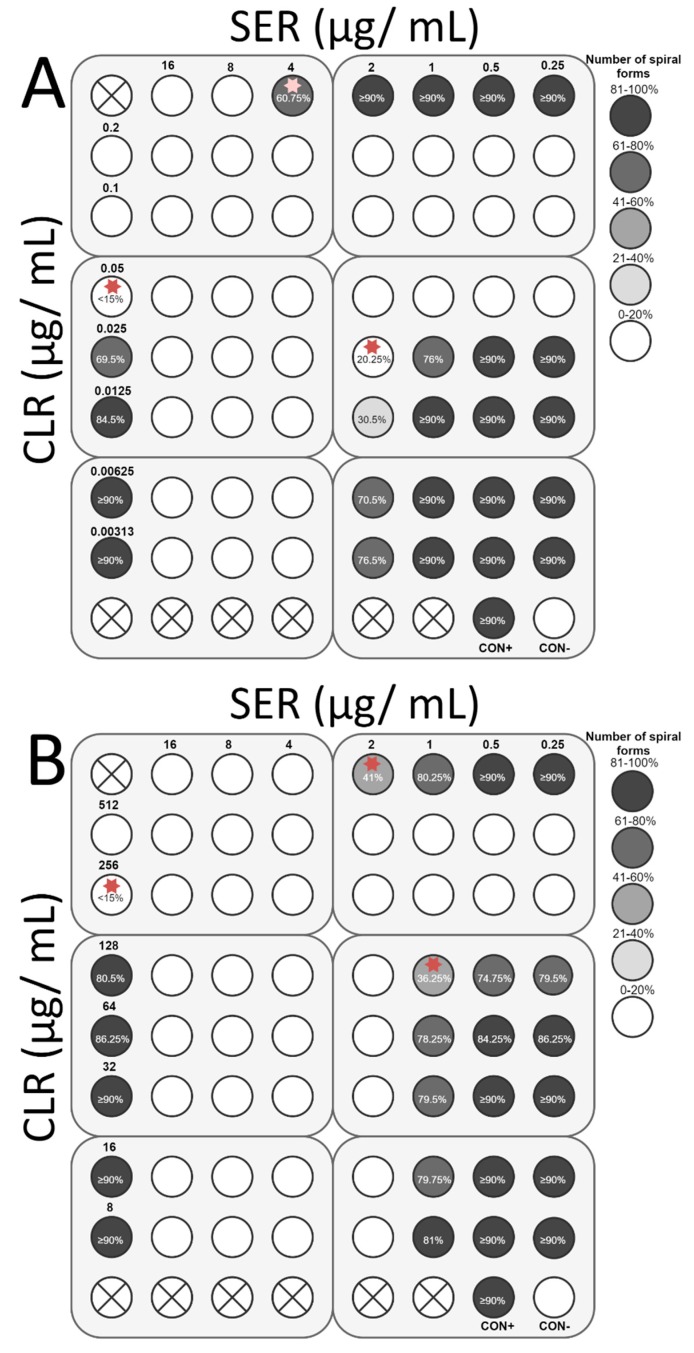
The antibacterial and morphological effect of sertraline (SER), clarithromycin (CLR), and combinations of both against *H. pylori* Tx30a and 7143 strains. The existence of interaction in the antimicrobial activity of SER with CLR was determined against the reference, antibiotic-susceptible *H. pylori* Tx30a (**A**) and the clinical, double-resistant *H. pylori* 7143 strain (**B**). The white circles indicate wells in which the number of spiral forms was ≤15%, while the white circles with a cross in the middle indicate empty wells. Using red asterisks, the wells with the MICs of the tested substances were marked, whereas in the case of the interactions verification, they indicate the lowest FIC. Abbreviations: Sertraline, SER; Clarithromycin, CLR; Positive control, CON+; Negative control, CON-.

**Figure 11:**
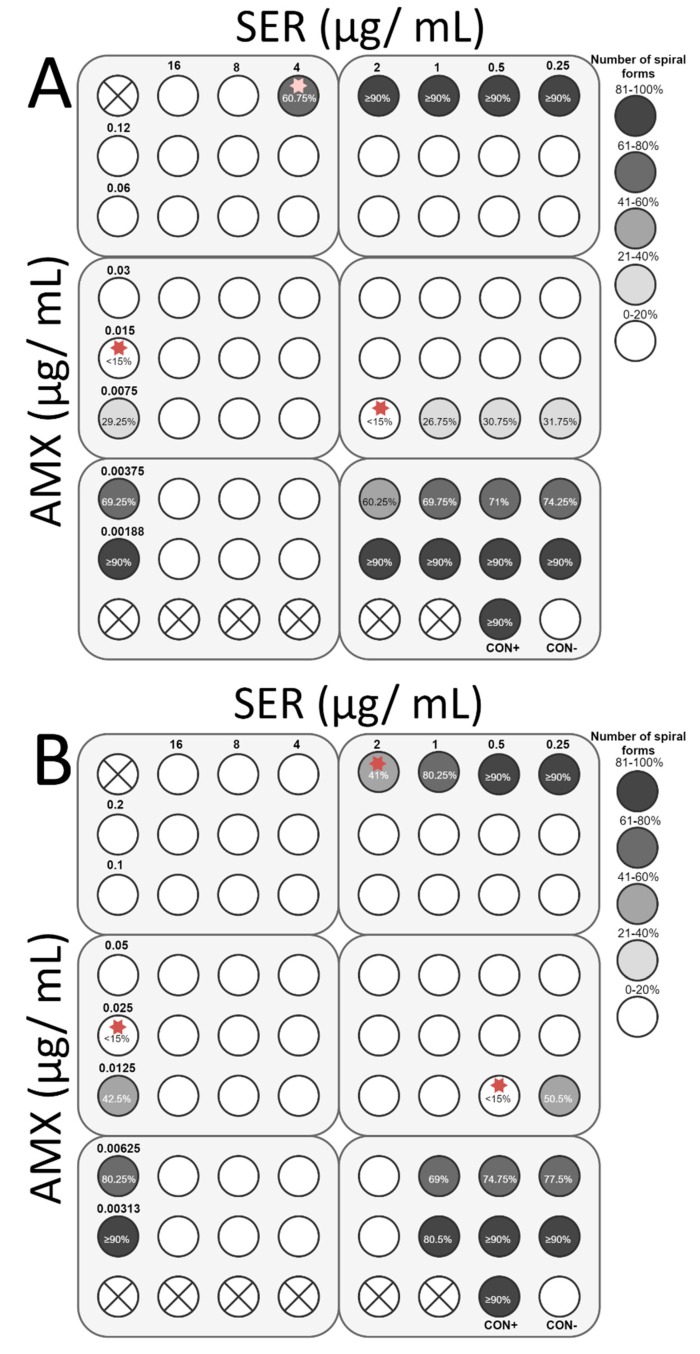
The antibacterial and morphological effect of sertraline (SER), amoxicillin (AMX), and combinations of both against *H. pylori* Tx30a and 7143 strains. The existence of interaction in the antimicrobial activity of SER with AMX was determined against the reference, antibiotic-susceptible *H. pylori* Tx30a (**A**) and the clinical, double-resistant *H. pylori* 7143 strain (**B**). The white circles indicate wells in which the number of spiral forms was ≤15%, while the white circles with a cross in the middle indicate empty wells. Using red asterisks, the wells with the MICs of the tested substances were marked, whereas in the case of the interactions verification, they indicate the lowest FIC. Abbreviations: Sertraline, SER; Amoxicillin, AMX; Positive control, CON+; Negative control, CON-.

The final stage of the study was to determine the ability of SER to increase the antibacterial activity of antibiotics commonly used against *H. pylori*, i.e., TET, MTZ, CLR, and AMX. For all antibiotics tested, the interaction with SER was synergistic or additive. Synergy of action with TET (FIC = 0.375) was observed for both strains, which was associated with an 8-fold decrease in the TET concentration (0.1 to 0.0125 µg/mL and 0.4 to 0.05 µg/mL for *H. pylori* Tx30a and 7143 strain, respectively) and a 4-fold reduction of the SER concentration (4 to 1 µg/mL and 2 to 0.5 µg/mL for *H. pylori* Tx30a and 7143 strain, respectively) (Figure 8). In addition, for *H. pylori* 7143 (MTZ-resistant) a synergy of SER with MTZ (FIC = 0.5) was demonstrated, with a 4-fold reduction in the concentration of both substances required to inhibit growth (Figure 9A). An intense decline of the MTZ concentration (16-fold, from 256 to 16 µg/mL) was also seen when the SER concentration was 2-fold reduced (FIC = 0.506, additivity), instead of a 4-fold decrease (Figure 9A). For *H. pylori* Tx30a (MTZ-sensitive), the interaction of MTZ with SER was additive (FIC = 1) (Figure 9B). For both strains, combinations of CLR with SER (FIC = 1 for both) or AMX with SER (FIC = 1 and FIC = 0.75 for *H. pylori* Tx30a and 7143 strain, respectively) were additive (Figure 10 and Figure 11).

In addition to determining bactericidal effect of both SER and antibiotics, the morphology of *H. pylori* exposed to tested substances was also measured (Supplementary Tables S11–S18). It has been noticed that SER is a weak stimulator of the development of coccoid forms, which was associated with maintaining a relatively high number of spiral forms at MIC values, counting for 60.75% and 41% for *H. pylori* Tx30a and 7143 strain, respectively (Figure 8, Figure 9, Figure 10 and Figure 11). At subinhibitory concentrations of SER, the number of spiral forms was ≥90% for *H. pylori* Tx30a. For *H. pylori* 7143, these numbers were equal to 80.25% and ≥90% when exposed to ½× MIC and ¼× MIC, respectively (Figure 8, Figure 9, Figure 10 and Figure 11). On the other hand, the domination of spherical forms was almost complete (number of spiral forms <15%) at MBCs of SER (Supplementary Table S11–S18). Unlike SER, MIC values of antibiotics contributed to the formation of a high number of coccoid forms (>85%) (Figure 8, Figure 9, Figure 10 and Figure 11). The most intensive inducer of the *H. pylori* morphological transition was AMX (at ½× MIC, 29.25% and 42.5% of spiral forms were detected for *H. pylori* Tx30a and 7143 strain, respectively). The weakest inducers of morphological changes were CLR relative to *H. pylori* 7143 (80.5% of spiral forms using ½× MIC) and MTZ relative to *H. pylori* Tx30a (75.5% of spiral forms using ½× MIC) (Figure 8, Figure 9, Figure 10 and Figure 11).

## 4. Discussion

Antibiotic resistance is a topic that has been intensively discussed in recent years. It has been noted that antibiotic resistance in *H. pylori* has reached an alarmingly high level worldwide [8]. Therefore, there is a need to find new substances with an activity directed against this bacterium [9]. The purpose of this article was to determine the impact of SER, a representative of SSRIs, on clinical and reference *H. pylori* strains. MICs and MBCs were found to be in the range of 2-8 µg/mL and 4-8 µg/mL, respectively. The concentrations obtained coincide with the values obtained for other microorganisms, including fungi [15,17,19,22,23,46] and Gram-positive bacteria [12,30]. It was noticed that Gram-negative bacteria show a lower sensitivity to SER, with the exception of *Moraxella catarrhalis*, *Brucella* spp., and *Campylobacter jejuni* [12]. For *C. jejuni*, a spiral Gram-negative rod that is closely related to *H. pylori*, the minimum concentrations inhibiting 50% of the tested isolates (MIC_50_) counted for 4 µg/mL [12]. This may suggest similarities in the structure/physiology of cells, affecting the higher sensitivity of these bacteria to SER.

The current study also showed that the MBC/MIC ratio is ≤2, with the exception of *H. pylori* 7143 strain, for which it was equal to 4. This observation suggests the bactericidal activity of SER against *H. pylori* [47]. Confirmation of the obtained result was made by a time-killing assay during which the concentration-dependent and time-dependent activity of this substance was noticed. In the course of the experiment, the number of spiral forms decreased with the proportional increase of spherical forms. Many in vitro studies have shown the presence of coccoid forms of *H. pylori* when this bacterium has been exposed to sublethal concentrations of antibiotics [48,49,50,51,52,53]. For these morphological forms, an involvement in therapeutic failure is suggested, which is related to an intensified biofilm-forming capability and increased expression of efflux pumps [54,55,56]. Therefore, substances limiting stimulation of morphological transformation of *H. pylori* are valuable from a therapeutic point of view. In the present study, it was observed that the effect of induction of spherical forms was closely associated with the concentration of SER used. Subinhibitory concentrations of this compound had a low potential to induce coccoid forms (4.5% and 16.5% of spherical forms after a 24-h incubation with ½× MIC for *H. pylori* Tx30a and J99, respectively). When using MIC, the stimulating effect of SER depended on the MBC value, because for *H. pylori* Tx30a (MBC = 2× MIC) a low number of spherical forms (27% after 24 h) was shown, while for *H. pylori* J99 (MBC = MIC) these morphotype was dominant (83.5% after 24 h). Despite the high numbers of coccoid forms in the SER-treated samples, this morphological transformation was not protective, as shown by fluorescence microscopy. Bacterial exposure to MBC and 2× MBC contributed to a 4-8 fold reduction in the green/red fluorescence ratio compared to the control. The high bactericidal activity of SER against *H. pylori* may be associated with the presence of different targets for this compound and limited possibilities for developing resistance. In other pathogenic organisms, the ability of SER to damage the cell membrane [24], inhibit protein translation [17], reduce the amount of cytoplasmic ATP [26,27,29], lower intracellular pH [36], and interfere with metabolism (including tricarboxylic acid cycle) has been observed [27,29]. The effect of SER on *H. pylori* is not known, but these reports may be the starting point in future studies determining the molecular changes in the SER-treated cells of this microorganism.

The final stage of the current study was to check the interaction between SER and antibiotics with an anti-*H. pylori* activity. Despite the diverse mechanism of action of these substances on microorganisms (TET, a 30S ribosomal subunit inhibitor [57]; CLR, a 50S ribosomal subunit inhibitor [58]; AMX, an antibiotic disrupting cell wall synthesis [59]; and MTZ, a genotoxic substance [60]), in the case of all a synergistic/additive interaction with SER was found. The non-selective, positive interaction of SER with many fungicides, including polyenes [15,18,61], echinocandins [15], and azoles [15,17,19,20,23], was also observed in experiments conducted on fungal microorganisms. The ability of this substance to increase the activity of structurally different substances may be related to the potential of SER to inhibit efflux pumps [36,37]. In *E. coli*, the synergistic activity of SER with TET has been shown, which was associated with a disturbance of the proton-motive force and an accumulation of TET [36]. The present study also showed the synergy of these substances (FIC = 0.375) with respect to both tested *H. pylori* strains. In addition, for the MTZ-resistant *H. pylori* 7143 strain, but not the MTZ-sensitive *H. pylori* Tx30a, a synergism of SER with MTZ (FIC = 0.5) was noticed. The reason for this phenomenon is unknown. It seems that this positive interaction may be associated with the ability of SER to inhibit protein translation [17]. In strains of *H. pylori* resistant to MTZ, an overexpression of superoxide dismutase (SodB), a protein reducing oxidative stress [62,63], is observed. Therefore, the use of an inhibitor disrupting the SodB activity may enhance an antibacterial effect of MTZ against these bacteria [64]. For other antibiotics, an additivity with SER may be related to an interference with efflux pumps and thus a sensitization of *H. pylori* to these substances. The participation of efflux pumps in antibiotic resistance of *H. pylori* has been demonstrated in many studies [65,66,67,68,69,70,71,72,73], while the use of efflux pump inhibitors (carbonyl cyanide m-chlorophenylhydrazone and Phe-Arg-beta-naphthylamide) correlated with an increased sensitivity to antibiotics [66,69,70,73].

Drugs with an antidepressant activity are a good example of substances that can be used in a drug repurposing approach. It is associated with the knowledge of potential side effects, toxicity, and pharmacokinetics of these compounds [74]. SER has a good tolerability profile [14,75]. Potential side effects include weight loss, decreased libido, gastrointestinal bleeding, or neurological ailments (fatigue, dizziness), but these usually resolve when continuing therapy [14,76]. It is indicated that SER is the best tolerated compound among SSRIs because it does not induce electrocardiographic changes and has a lower chance of non-adherence [14,75]. Normally, SER is taken in the form of tablets, capsules or oral concentrates, with a bioavailability directly proportional to the dose consumed [75]. Due to the slow half-life, counting for about 26 h, it is possible to take one dose daily [14,75]. SER has favorable physicochemical properties because it has been observed that its solubility increases at low pH [77,78] and a 3-h incubation of SER with 5 N HCl or NaOH solutions did not affect the stability of this compound [78]. Therefore, it seems that the stomach environment will not adversely affect the activity and dosage of SER. 

SER has been shown to achieve much higher levels in organs than in plasma [79,80]. For example, the SER concentration was 74-fold, 67-fold, and 22-fold higher in the liver, lungs, and brain, respectively [79]. The concentration of SER in the stomach is almost 10 times higher than in blood [80]. Such SER properties may translate into the high therapeutic efficacy of this compound against microorganisms in vivo. This was confirmed by the Rhein et al., who showed that despite low blood levels (0.2 µg/mL and 0.4 µg/mL for 200 and 400 mg/day, respectively) SER accumulates in the brain, reaching much higher concentrations. The use of SER in a dose of 200 mg/day allowed for the destruction of 65% of *Cryptococcus neoformans* strains (MIC = 1–8 µg/mL) and 90% when co-treated with fluconazole [46]. Similarly, 10-fold higher concentrations of SER in the stomach would allow to reach concentrations of about 2 µg/mL at the 200 mg/day dose. This, together with the intake of other antibiotics lowering the MIC of SER by 2–4 times, would result in obtaining MBC values against *H. pylori* in the gastric environment. In addition, bile with a 36-fold higher SER concentration [79] can be an independent factor that increases the concentration of this compound in the stomach. A similar mechanism promoting increased concentrations of antimicrobial substances in gastric juice is present in the case of AMX, an antibiotic routinely used in the therapy of *H. pylori* [81,82]. 

## 5. Conclusions

This research is the first, according to the authors’ knowledge, indicating the antibacterial activity of SER against *H. pylori*, which was independent from its antibiotic resistance profile. It was observed that subinhibitory concentrations of this compound did not stimulate the formation of antibiotic-tolerant coccoid forms. On the other hand, these bacteria did not survive treatment with MBCs, regardless of the morphology presented. In addition, SER has been shown to potentiate the activity of all four antibiotics tested (AMX, CLR, TET, and MTZ). These results indicate that SER may be a promising anti-*H. pylori* compound.

## Figures and Tables

**Table 1:** The minimal inhibitory concentrations (MICs) and minimal bactericidal concentrations (MBCs) of sertraline (SER) against selected *H. pylori* strains.

Strains	Antibiotic Resistance *		Activity of SER		
	MET	CLR	MIC **	MBC **	MBC/MIC Ratio
J99	S	S	4	4	1
Tx30a	S	S	4	8	2
6237	S	S	4	4	1
7471	S	S	8	8	1
7189	S	R	4	8	2
7556	S	R	2	4	2
7388	R	S	4	4	1
7394	R	S	4	8	2
7143	R	R	2	8	4
7649	R	R	4	4	1

* Antibiotic resistance: S, Sensitive; R, Resistant; MET, Metronidazole; CLR, Clarithromycin. ** The MIC and MBC values are given in µg/mL.

## Supplementary Materials

https://www.mdpi.com/2076-0817/8/4/228/s1. Table S1. Zones of inhibition (mm) of sertraline (SER) against antibiotic-susceptible *H. pylori* strains. Table S2. Zones of inhibition (mm) of sertraline (SER) against metronidazole-resistant *H. pylori* strains. Table S3. Zones of inhibition (mm) of sertraline (SER) against clarithromycin-resistant *H. pylori* strains. Table S4. Zones of inhibition (mm) of sertraline (SER) against CLR- and MTZ-resistant *H. pylori* strains. Table S5. Effect of sertraline (SER) on the culturability of *H. pylori* Tx30a. Table S6. Effect of sertraline (SER) on the culturability of *H. pylori* J99. Table S7. Effect of sertraline (SER) on the number of spiral forms of *H. pylori* Tx30a. Table S8. Effect of sertraline (SER) on the number of spiral forms of *H. pylori* J99. Table S9. Analysis of mean green/red fluorescence ratios during the incubation of *H. pylori* Tx30a with sertraline (SER) in time. Table S10. Analysis of mean green/red fluorescence ratios during the incubation of *H. pylori* J99 with sertraline (SER) in time. Table S11. The morphology of *H. pylori* Tx30a after 3-days incubation with SER and CLR during the checkerboard assay. Table S12. The morphology of *H. pylori* Tx30a after 3-days incubation with SER and TET during the checkerboard assay. Table S13. The morphology of *H. pylori* Tx30a after 3-days incubation with SER and AMX during the checkerboard assay. Table S14. The morphology of *H. pylori* Tx30a after 3-days incubation with SER and MTZ during the checkerboard assay. Table S15. The morphology of *H. pylori* 7143 after 3-days incubation with SER and CLR during the checkerboard assay. Table S16. The morphology of *H. pylori* 7143 after 3-days incubation with SER and TET during the checkerboard assay. Table S17. The morphology of *H. pylori* 7143 after 3-days incubation with SER and AMX during the checkerboard assay. Table S18. The morphology of *H. pylori* 7143 after 3-days incubation with SER and MTZ during the checkerboard assay. Figure S1. Fluorescence microscopy of *H. pylori* Tx30a during the incubation with sertraline (SER). Figure S2. Fluorescence microscopy of *H. pylori* J99 during the incubation with sertraline (SER).
